# Current trends in small molecule discovery targeting key cellular signaling events towards the combined management of diabetes and obesity

**DOI:** 10.6026/97320630013394

**Published:** 2017-12-31

**Authors:** Kadapakkam Nandabalan Sangeetha, Sundaresan Sujatha, Velusamy Shanmuganathan Muthusamy, Singaravel Anand, Kusampudi Shilpa, Posa Jyothi kumari, Baskaran Sarathkumar, Gopal Thiyagarajan, Baddireddi Subhadra Lakshmi

**Affiliations:** 1 Department of Biotechnology, Anna University, Chennai Tamilnadu, India - 600 025; 2 Centre for Food Technology, Department of Biotechnology, Anna University, Chennai Tamilnadu, India - 600 025

**Keywords:** Diabetes, Obesity, PTP1B inhibitors, PPAR modulators, Natural products

## Abstract

Non-insulin dependent diabetes mellitus, also known as Type 2 diabetes is a polygenic disorder leading to abnormalities in the carbohydrate and lipid metabolism. The major contributors in the pathophysiology of type 2 diabetes (T2D) include resistance to insulin action, β cell dysfunction, an abnormality in glucose metabolism and storage, visceral obesity and to some extent inflammation and oxidative stress. Insulin resistance, along with a defect in insulin secretion by the pancreatic β cells is instrumental towards progression to hyperglycemia. Increased incidence of obesity is also a major contributing factor in the escalating rates of type 2 diabetes. Drug discovery efforts are therefore crucially dependent on identifying individual molecular targets and validating their relevance to human disease. The current review discusses bioactive compounds from medicinal plants offering enhanced therapeutic potential for the combined patho-physiology of diabetes and obesity.

We have demonstrated that 3β-taraxerol a pentacyclic triterpenoid (14-taraxeren-3-ol) isolated from the ethyl acetate extract of Mangifera indica, chlorogenic acid isolated from the methanol extract of Cichorium intybus, methyl tetracosanoate from the methanol extract of Costus pictus and vitalboside A derived from methanolic extract of Syzygium cumini exhibited significant effects on insulin stimulated glucose uptake causing insulin sensitizing effects on 3T3L1 adipocytes (an in vitro model mimicking adipocytes). Whereas, (3β)-stigmast-5-en-3-ol isolated from Adathoda vasica and Aloe emodin isolated from Cassia fistula showed significant insulin mimetic effects favoring glucose uptake in L6 myotubes (an in vitro model mimicking skeletal muscle cells). These extracts and molecules showed glucose uptake through activation of PI3K, an important insulin signaling intermediate. Interestingly, cinnamic acid isolated from the hydro-alcohol extract of Cinnamomum cassia was found to activate glucose transport in L6 myotubes through the involvement of GLUT4 via the PI3K-independent pathway. However, the activation of glucose storage was effective in the presence of 3β-taraxerol and aloe emodin though inhibition of GSK3β activity. Therefore, the mechanism of improvement of glucose and lipid metabolism exhibited by the small molecules isolated from our lab is discussed. However, Obesity is a major risk factor for type-2 diabetes leading to destruction of insulin receptors causing insulin resistance. Identification of compounds with dual activity (anti-diabetic and antiadipogenic activity) is of current interest. The protein tyrosine phosphatase 1B (PTP1B) is an important negative regulator of the insulin and leptin-signaling pathway is of significance in target definition and discovery.

## Background

Deregulation of biological pathways leads to a host of diseases in human. Thus, identification of molecules as drugs that modulates the impaired molecular targets is of importance despite considerable progress over decades. This is even more challenging in combating multi-genic diseases such as cancer, diabetes and inflammatory disorders. In such cases, modulating the activity of multiple targets is essential to achieve optimal therapeutic benefit [1]. Hence, a single drug or a combination of drugs that simultaneously impact multiple targets are preferred for control of complex disease systems. [2].

T2D is a multi-factorial disorder resulting in abnormalities of carbohydrate and lipid metabolism [3]. The incidence of T2D is rapidly becoming a global pandemic and has been projected to afflict more than 300 million individuals worldwide by the year 2025 [4]. The epidemic of diabetes with its associated complications has a huge impact on healthcare as well as human morbidity and mortality. Although the primary cause of this disease is unknown, it is clear that insulin resistance plays an early role in its pathogenesis. Insulin resistance occurs via multiple mechanisms and mediators. Among them, elevated free fatty acid (FFA) levels have been reported as a key cause of insulin resistance along with an altered glucose output and uptake [5]. Increased levels of FFA decrease insulin sensitivity via impairing the anti-oxidant defense by generation of reactive oxygen species, and an over expression of inflammatory markers [6]. Elevated FFAs lead to a reduction in intracellular glutathione levels [7] and along with inflammation and oxidative stress are the factors that implicate adipo-genesis and insulin resistance.

## NON-INSULIN DEPENDANT DIABETES MELLITUS (NIDDM) management

Our current understanding of NIDDM has helped identify several potential and specific targets for the management of NIDDM and its related secondary disorders. There are ranges of molecular drug targets identified on the basis of modulation of one or more key aspects of the pathogenesis of diabetes [8]. These include (i) reduction in the excessive hepatic glucose production (Glucagon, Hexokinase, G6P, F16BP, GSK3β); (ii) increased glucose-stimulated insulin secretion (DPP-IV inhibitors, GLP-1 and GLP-1 receptor agonists); (iii) inhibition of specific molecular targets involved in insulin signaling pathway (resistin, PTP1B inhibitors and SHIP2 inhibitors); (iv) control of obesity and altered lipid metabolism (Adiponectin, AMPK and PTP1B inhibitors).

It should be noted that targets that control carbohydrate as well as lipid metabolism are gaining increased attention in recent years. PTP1B is the known key enzyme that attenuates insulin transduction cascade by desensitizing the insulin receptor [9]. Since diabetes is a multi-genic disorder, drugs designed to act against individual molecular targets cannot be effective. Hence, successful strategies for control of the metabolic disorder would require a better mechanistic understanding followed by development of drugs (either single or in combination) that impacts multiple targets simultaneously. The combination drugs currently employed are primarily of rational design, but the increased efficacy they provide justifies in vitro discovery efforts for identifying novel multi-target mechanisms. This review presents a systematic approach towards identifying interactions between molecular pathways that could be leveraged for the management of diabetes and obesity using several phytoactive principles; thereby offering enhanced therapeutic potential with broader specificity on multiple targets.

## Drug templates for diabetes and obesity from plants

Natural products and their derivatives traditionally serve as therapeutic agents due to their structural diversity, biochemical specificity and maximum therapeutic efficacy with minimum side effects [10]. Most of these active principles originate from edible plants, so their inclusion in diet would undoubtedly be of value due to their hypoglycemic potential. Several phytomolecules including flavonoids, alkaloids, glycosides, saponins, glycolipids, dietary fibres, polysaccharides, peptidoglycans, carbohydrates, amino acids, triterpeniods, steroids, xanthone, coumarins, iridoids, alkyl disulphides, inorganic ions and guanidines obtained from various plant sources have been reported to exhibit anti-diabetic activity [11].

The anti-diabetic effect of six different plants Cichorium intybus, Cinnamomum cassia, Costus pictus, Mangifera indica, Adathoda vasica, Cassia fistula and Syzygium cumuni, the bioactive molecules isolated through bioactivity-guided fractionation is reviewed. Chlorogenic acid (CGA), a major hydrolysable tannin was isolated from methanolic extract of Cichorium intybus, cinnamic acid was isolated from the hydro-alcoholic extract of Cinnamomum cassia, methyl tetracosanoate (MT), a saturated fatty acid was isolated from the methanol extract of Costus pictus, 3β- taraxerol a pentacyclic triterpenoid (14-taraxeren-3-ol) was purified from ethyl acetate extract of Mangifera indica, (3β)- stigmast-5-en-3-ol is a triterpenoid isolated from the ethyl acetate extract of Adathoda vasica, Aloe emodin-8-O-glycoside (AEG), an anthroquinone from the methanol extract of Cassia fistula and Vitalboside A (VBA) or Oleanolic acid-3-O-glucoside, a pentacyclic triterpene glycoside was isolated from the methanolic extract of Syzygium cumuni (Table 1).

## Insulin sensitisers

Insulin sensitivity is determined by the ability of insulin to promote glucose uptake and utilization. Insulin activates a series of signal transduction events that results in specific biological action on insulin sensitive tissues. Insulin binds to the insulin receptor leading to auto-phosphorylation of the receptor through its tyrosine kinase activity and, subsequent phosphorylation of insulin receptor substrates (IRS). Phosphorylation of IRS leads to activation of the phosphatidyl-inositol 3-kinase (PI3K) pathway, which plays an important role in glucose transport and disposal. Insulin resistance is an impairment of the signaling cascade that results in inappropriate glucose and lipid metabolism. The most conclusive evidence for defective insulin sensitivity in type 2 diabetes comes from euglycemic and hyperinsulinemic clamp studies, in which the total body glucose clearance is shown to be reduced in type 2 diabetic subjects [12]. Furthermore, the primary site of reduced insulin-mediated glucose uptake is located in the peripheral muscle tissue [13, 14]. Thus, insulin insensitivity described as insulin resistance with its associated abnormalities has become a major challenge and has prompted pharmaceutical companies to search for new insulin sensitizing agents.

3β-taraxerol, a pentacyclic triterpenoid (14-taraxeren-3-ol) purified from Mangifera indica has been observed to show insulin sensitizing action in an in vitro adipocyte model. The sensitizing effect of the natural compound was revealed through its significant effect on insulin stimulated glucose uptake, in an IRTK/PI3K dependant activation that facilitated an effective GLUT4 translocation and activation [15]. Methyl tetracosanoate (MT) exhibited its promising anti-diabetic activity through its insulin sensitizing action by activation of key targets of insulin signaling cascade in a similar manner [16]. Vitalboside A, a small molecule derived from Syzygium cumini showed activity as an insulin sensitizer by increasing glucose uptake in both differentiated L6 myocytes and 3T3-L1 adipocytes through PI3K/AKT signaling pathway [17]. TZDs are anti-diabetic drugs that enhance insulin sensitivity and so the insulin sensitizing effects of compounds discussed in this review were validated with a derivative of TZD - Rosiglitazone. Since these biomolecules are known to modulate the insulin signal transduction pathway their activity could be therapeutically relevant.

We have also postulated that cinnamic acid activates glucose transport in L6 myotubes independent of PI3K activation [18]. Adisakwattana et al. [19] have reported the insulin secretagogue effect of cinnamic acid and its derivatives. This dual activity of cinnamic acid on both insulin signaling and secretion reveals it as an ideal prototypic compound for structural and functional studies.

## Insulin mimetics

Another class of compounds that improves insulin action is the insulin mimickers. They act similar to insulin and exhibit their effects on insulin sensitive tissues independent of insulin stimulation. An uptake of glucose into the insulin targeted tissues is one of the mechanisms implicated in the blood glucose lowering effect of these agents, which seems to result from beneficial extra-pancreatic actions on glucose clearance rather than from enhanced insulin release [20]. Sujatha et al. have reported (3β)-stigmast-5- en-3-ol, a plant phytosterol to possess insulin like effects [21]. This insulin mimetic effect of (3β)- stigmast-5-en-3-ol was initially established based on its efficacy in stimulating basal glucose uptake in differentiated L6 myotubes and, was later confirmed through molecular mechanistic studies on the targets involved in the insulin signaling cascade. A sequential activation of IR-β, IRS-1, PI3K, AKT/PKB, PKCα and GLUT4 were observed in rat skeletal muscle cells. Since the restoration of glucose uptake activity by (3β)-stigmast-5-en-3-ol was evidenced without stimulation of insulin, it led us to suggest the involvement of insulin-like property to be the mechanism underlying the in vitro anti-diabetic activity [21]. Wang et al. [22] have demonstrated the cholesterol-lowering efficacy of (3β)- stigmast-5-en-3-ol. Based on these evidences, we hypothesize that (3β)-stigmast-5-en-3-ol can be of interest as an anti-diabetic therapeutic, since it improves insulin resistance through its insulin mimetic property coupled with cholesterol lowering potential. Similarly, AEG exhibits insulin mimetic activity by upregulating glucose uptake irrespective of insulin stimulation through the activation of IR-β, IRS1, p85 subunit of PI3K, PKB and finally GLUT4 translocation [23].

## Activators of glucose storage

In T2D, insufficient action of insulin in liver and elevated circulating levels of glucagon could account for the high rate of hepatic glucose production, contributing to increased blood glucose levels [24]. Modulation of the production of hepatic glucose could be an important strategy towards inducing glucose storage through glycogen synthesis. Glycogen synthesis occurs by activation of the insulin-signaling pathway in which PI3K phosphorylates protein kinase B (Akt) that in turn inhibits GSK3β activity. These sequential events result in the activation of glycogen synthase, subsequently converting glucose to glycogen [25]. T2DM is associated with increased levels of GSK3β, and inhibitors of its activity can reproduce the effects of insulin. Therefore, inhibition of GSK3β might represent a potential target to enhance glycogen synthesis. Although, there are very few agents that have an effect on glycogen stores (Metformin), they still possess the risk of developing side effects like hypoglycemia. 3β-taraxerol and AEG two structurally dissimilar compounds of different origin have been reported to induce glycogen synthesis independent of insulin stimulation. Both the compounds showed significant inhibitory effects on GSK3β activity through activation of PKB [15, 23]. Likewise, MT a saturated fatty acid also exhibited significant inhibition of GSK3β activity [16].

## Inhibitors of adipogenesis

The progression of diabetes and its associated complications has been observed to increase with obesity. However, currently available anti-diabetic drugs like Thiazolidinediones and Sulphonylureas promote obesity while reducing blood glucose levels. Hence, drugs that regulate carbohydrate metabolism without disturbing lipid metabolism are the current need of the hour [26]. As herbal preparations are anticipated to offer strong therapeutic potential, we have attempted to address this issue with Cichorium intybus, a salad crop. Since the methanol extract of the plant (CME) promoted glucose transport in a PI3K dependant manner combined with inhibition of adipocyte differentiation in 3T3L1 adipocytes, we attempted to isolate the bioactive principle responsible for the dual activity. As tannins formed the major composition of the extract the role of tannins present in the methanol extract was evaluated after detannification (CME/DT). But surprisingly the activity exhibited by the detannified extract was exactly vice versa to the methanol extract [27]. This interesting observation coincides with early reports on inhibitory role of tannins in adipocyte differentiation [28].

Peroxisome proliferator activator receptor (PPAR), CCAAT/enhancer binding protein (C/EBP), and sterol regulatory element binding protein (SREBP) families are the welldocumented primary adipogenic transcription factors involved in adipocyte differentiation. Among them PPARγ is most extensively studied for its therapeutic utility in the treatment of NIDDM [29]. The methanol extract of Cichorium intybus (CME) controls the expression of all the three adipogenic markersand the molecular mechanisms of the anti obesity and anti diabetic effects of flavonoids have been well studied [30]. The detannified extract (CME/DT), however did not exhibit an inhibitory activity [27]. Based on this Chlorogenic acid (CGA) was isolated from CME [27]. Despite isolating the pure molecule (CGA) after enrichment from the crude extract (CME) there was no significant enrichment in adipogenesis inhibition [27]. Wagner et al has reported that the therapeutic superiority of crude extract over an isolated single molecule would be caused due to additive effects of the other molecules present in the extract [31]. The plant C. intybus has been reported for the presence of cichoric acid (CHA), traces of caffeic acid (CFA) and cinnamic acid [32, 33]v. Hence, we hypothesize that association of CFA and CHA would be adding to the net biological activity of CME. This observation substantiates the earlier hypothesis on higher efficacy of tannins present in CME than the non-tannins present in CME/DT (with PPARγ agonism).

Similarly, the methanol extract of Costus pictus has been reported to possess dual activities like anti-diabetic and anti-adipogenic activities. But the identified bioactive principle methyl tetracosanoate (MT) did not exhibit adipogenic inhibitory effect [16]. The prospect of identifying the anti-adipogenic molecule from CPME favoring treatment options for obesity-induced diabetes exists. We have also reported Aloe emodin-8-Oglycoside (AEG), a plant derived anthroquinone to possess a promising effect on adipogenesis inhibition thus highlighting the importance of it as a glucose-lowering agent without inducing adipogenesis [23]. The methanolic extract of Syzygium cumini (SCME) and its dual bioactive compound, Vitalboside A (VBA) were found to exhibit both anti-diabetic and anti-adipogenic effect. Interestingly, VBA inhibited partial PPARγ2 transcriptional activity in transiently transfected HEK-293 cells resulting in partial inhibition of lipid accumulation in adipocytes without affecting synthesis and secretion of adiponectin, [17]. Further, investigation of the in vivo efficacy of SCME on insulin resistance in high fat diet (HFD) induced obese C57BL/6J mice showed significant reduction in insulin resistance, dyslipidemia and body weight gain with concomitant increase in glucose tolerance. Elucidation of bifunctionality of SCME was found to exhibit inhibition of PTP1B and partial activation of PPARγ activity in skeletal muscle and visceral adipose tissue of HFD fed obese mice.

## PTP1B inhibitors

Targeting the common form of insulin resistance in type 2 diabetes is difficult since it involves multiple non-specific insulin signaling defects [3]. Usually, most intracellular signaling takes place via cascades of phosphorylating enzymes (kinases) and the de-phosphorylation reactions are catalyzed by protein phosphatases. Protein tyrosine phosphatases (PTPs) belong to the family of classical and dual specificity phosphatases and, have emerged as new and promising class of signaling targets since the discovery of PTP1B [34]. Protein tyrosine phosphatases (PTPases), act as physiological negative regulator of insulin signaling by dephosphorylating the activated insulin receptor (IR), thereby limiting the insulin-signaling pathway. An increased expression and activation of PTPases has been observed in muscle and fat from rodents, obese and diabetic humans and are believed to be involved in the pathogenesis of insulin resistance [3]. PTP deleted mice have been reported to be healthy, lean and obesity resistant with reduced levels of insulin and glucose. This phenotype has been ascribed to restore insulin sensitivity through insulin receptor (IR) and insulin receptor substrate (IRS1) in the absence of PTP1B, thus projecting the importance of PTP1B as drug target for diabetes and obesity [35][36] . PTPs have control over both leptin and insulin signaling [37], therefore inhibition of PTPase activity seems to be an appealing strategy for obesity and diabetes.

We have also isolated bioactive molecules from several plants with potential PTP1B inhibitory effect. Chlorogenic acid (CGA) was isolated from C. intybus through bioactivity-guided purification and reported by our group [27]. It is an ester formed between caffeic acid and (L)-quinic acid and is a major hydrolysable tannin compound present in coffee [38]. CGA has earlier been reported for glucose uptake [39], which was later, confirmed by Muthusamy et al [27]. Rodinone et al [40] observed a decrease in adipogenesis with a down regulation of all adipogenic genes in animals ablated with PTP1B, and postulated the pivotal role of PTP1B in the development of obesity. Similarly, we have reported that the PTP1B inhibitory potential of CGA would be the linking factor that is responsible for the observed increase in glucose uptake and decreased adipogenesis [27]. Analysis of CGA and other caffeoyl derivatives of the methanolic extract of cichory salad leaves were found to exhibit better insulin sensitivity in both in vitro and in vivo experiments through inhibition of PTP1B [41]. Our study further confirmed the non-competitive binding nature of CGA and CHA, which was established by co-incubating it with sodium orthovanadate (SOV) a protein tyrosine phosphatase inhibitor [27]. Vanadate based inhibitors inhibit PTPs non-selectively and have been used with some success in clinical trials with only moderate toxicity. This suggests that PTP1B inhibiting drug need not have absolute selectivity [34]. Computational approaches to study the binding and interaction of these two compounds at the Allosteric site of PTP1B located ~20 A away from the catalytic site was performed using molecular docking and MD simulations. Results showed signification allosteric interactions and modulation at the allosteric alpha 7 helix restraining the activation of the protein, in comparison to Compound-2, a standard allosteric inhibitor [42]. However, recent experimental evidence using NMR- chemical shift analysis accompanied with docking and MD simulations show a non-competitive binding of CGA and a competitive inhibition by CHA [43]. Although, this could be due to the fact that these compounds were not subjected to co-incubation assay with a strong catalytic inhibitor such as SOV, it requires further investigation.

We have also reported the PTP1B inhibitory potential of 3β- taraxerol, a triterpenoid from Mangifera indica and Methyl tetracosanoate (MT), a saturated fatty acid from Costus pictus in comparison to SOV [15, 16, 44]. The Aloe emodin glycoside (AEG) isolated from Cassia fistula was observed to exhibit a moderate effect on PTP1B inhibition eliminating the possibility of AEG as a potent PTP1B inhibitor [23]. The methanolic extract of Syzygium cumini (SCME) and the dual active molecule Vitalboside A (VBA) it showed a dose-dependent inhibition of PTP1B. Molecular docking and co-incubation studies show them to selectively inhibit PTP1B at its allosteric site [17]. Thus, it is evident that compounds with different structures isolated from plant species can act as active moieties and serve as templates for the development of PTP1B inhibitors.

## Conclusion

Our continued interest to identify therapeutics for diabesity has resulted in the isolation of several active principles from different plant sources. These principles are structurally dissimilar with different characteristic features and yet they exhibit significant biological activity. It is evident that each of the active moieties isolated so far have shown potential in leveraging multiple targets involved in diabesity with their unique mode of action towards commercial exploitation by pharmaceutical industry. Hence, it is prudent that further evaluation of the properties and applications of these compounds will greatly enhance their perspectives in drug discovery.

## Figures and Tables

**Table 1:** List of potential anti diabetic and anti adipogenic compounds isolated from Plants

Plant name	Bioactive molecule	Activity	Reference
Adathoda vasica	(β)-stigmast-5-en-3-ol	Insulin mimetic	Sujatha et al. 2010 [21]
Cassia fistula	Aloe emodin glycoside	Insulin mimetic and anti -adipogenic	Anand et al. 2010 [23]
Cichorium intybus	Chlorogenic acid	Anti-diabetic and anti-adipogenic	Muthusamy et al. 2008 [27]
		Allosteric inhibition towards PTP1B	Sarath Kumar et al. 2012 [42]
Cinnamomum cassia	Cinnamic acid	Insulin signaling independent of PI3K	Lakshmi et al. 2009 [18]
Costus pictus	Methyl tetracosanoate	PTP1B inhibition and PI3K activation	Shilpa et al. 2009 [16]
Mangifera indica	3β-taraxerol	Glucose transport and storage	Sangeetha et al. 2010 [15]
Syzygium cumini	Vitalboside A	PTP1B inhibitor and partial PPARγ agonist with adiponectin secretagogue	Thiyagarajan et al. 2016 [17]
